# Synthesis and characterization of bromo-functional acrylate polymers

**DOI:** 10.1039/d6ra02373h

**Published:** 2026-07-23

**Authors:** Ülfet Akgün, Arzu Hatipoğlu, Tarik Eren

**Affiliations:** a Department of Chemistry, Yildiz Technical University Istanbul Türkiye teren@yildiz.edu.tr

## Abstract

Acrylates are among the most widely utilized monomers in industrial polymer chemistry, serving as key components in coatings, adhesives, and high-performance polymer matrices. Brominated compounds are well recognized for their ability to inhibit or retard flame propagation in organic materials; moreover, the C–Br bond serves as a versatile reactive handle for post-synthetic modification *via* nucleophilic substitution, enabling further functional diversification of the polymer structure. In this study, a series of novel bromo-functional acrylate polymers were synthesized and systematically characterized. The synthetic approach involved the conversion of cyclohexene to a brominated acrylate monomer *via* reaction with excess acrylic acid and *N*-bromosuccinimide (NBS), followed by free-radical polymerization initiated by azobisisobutyronitrile (AIBN). Subsequently, the resulting polymers were partially functionalized with 1,4-diazabicyclo[2.2.2]octane (DABCO) through quaternization, yielding partially DABCO-functionalized polymers. Additionally, the bromo-bearing polymers were reacted with sodium azide to introduce pendant azide (–N_3_) groups, enabling further structural modification through click-chemistry pathways. Both modification routes resulted in partial conversion of the bromine functionalities. The incorporation of bromine functionalities and their subsequent chemical transformations provide a versatile platform for the preparation of functional polymer derivatives. These materials may be further explored in future studies for applications such as antimicrobial coatings, ion-exchange membranes, and flame-retardant materials.

## Abstract 2

Novel bromo-functional acrylate polymers were synthesized *via* free-radical polymerization of a brominated acrylate monomer. The pendant C–Br groups provide a versatile platform for post-polymerization functionalization, enabling the development of advanced functional polymeric materials.

## Graphical abstract

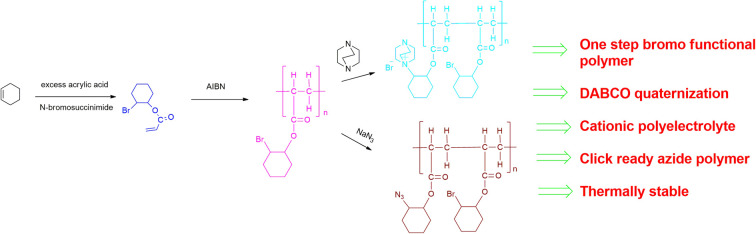

## 1. Introduction

Polymers attract ever-increasing interest in both academic and industrial settings, playing crucial roles in a broad spectrum of applications owing to their versatility, durability, and wide range of attainable properties.^1–4^ The global polymer market was valued at USD 716.83 billion in 2022 and is projected to reach approximately USD 1.21 trillion by 2032, with a compound annual growth rate (CAGR) of 5.4% from 2023 to 2032. The future of polymer usage will be defined by a focus on sustainability, advanced functionality, and seamless integration with cutting-edge technologies.^5^ Polymers will continue to be at the forefront of innovation across diverse industries, exhibiting specific functionalities such as self-healing behavior, flame retardancy, biocompatibility, biocidal activity, and electrical conductivity, thereby driving progress in healthcare, electronics, energy, and environmental protection. The judicious selection of functional monomers, or the introduction of functional groups onto the polymer backbone, constitutes the primary strategy for imparting desired properties to end products. Functional side chains or pendant groups can be incorporated into the polymer backbone to endow it with tailored chemical and physical characteristics. Functional (meth)acrylates, in particular, exhibit a broad range of properties including rubbery, soft, and hard textures; optical transparency; mechanical flexibility; good heat resistance; the ability to withstand high temperatures and weathering; and glass transition temperatures below room temperature.

Brominated polymers are a class of macromolecules in which bromine atoms are incorporated into the polymer structure, typically to enhance properties such as flame retardancy. In the European Union, the use of certain brominated flame retardants (BFRs) is banned or restricted due to concerns about the risks these compounds pose to public health owing to their environmental persistence.^6^ As a consequence, there is growing demand for non-halogenated flame retardants based on phosphorus, nitrogen, or inorganic compounds such as aluminum hydroxide and magnesium hydroxide. Despite restrictions on certain bromine-based flame retardants, the bromine functional group remains a versatile and highly reactive site in organic and polymer chemistry, enabling the formation of diverse new functional groups through substitution, elimination, and addition reactions. Several comprehensive reviews discuss the modification of bromine-containing polymers with nucleophiles.^7–10^ Quaternization of alkyl bromides with amines or phosphines introduces cationic charge density, conferring biocidal activity to the resulting materials.^11^ Furthermore, brominated polymer networks have been shown to exhibit reversible redox peak potentials in cyclic voltammetry and are capable of quantitatively degrading hydrazine derivatives.^12^

Synthetic routes to bromine-functionalized polymers can be broadly divided into two categories: introduction of bromine during polymerization, and post-polymerization modification. Direct polymerization of vinyl bromide monomers *via* radical, anionic, or coordination mechanisms affords poly(vinyl bromide), in which bromine atoms are directly attached to the main chain. Over fifty years ago, Blauer *et al.* reported the synthesis of poly(vinyl bromide) using 2,2′-azobisisobutyronitrile as a thermal initiator, with no significant loss of bromine from the polymer under the conditions employed.^13^ The bromine-containing methacrylate monomer 2-(2-bromopropionyloxy)ethyl methacrylate (BPEM) has been incorporated into the formulation of radiopaque acrylic cements, simultaneously enhancing compressive strength and imparting radiopacity.^14^ Step-growth polymerization of brominated diols and diacids provides polyesters and polyamides with bromine atoms embedded in the backbone or side chains, yielding fire-resistant resins.^15,16^

Post-polymerization halogenation represents a complementary strategy. Polymers bearing reactive sites such as aliphatic double bonds, hydroxyl groups, or electron-rich aromatic rings can be brominated by treatment with molecular bromine (Br_2_)^17,18^ or *N*-bromosuccinimide (NBS).^19^ For instance, Zhou *et al.* synthesized well-defined brominated polymers through the bromination of poly(2-hydroxyethyl methacrylate) (PHEMA) with diethylaminodifluorosulfinium tetrafluoroborate (XtalFluor-E).^20^ Atom transfer radical polymerization (ATRP) using bromine-containing initiators or chain-transfer agents introduces terminal or pendant C–Br groups, which can subsequently serve as handles for controlled depolymerization in the presence of an iron catalyst.^21^

Despite extensive research on brominated vinyl monomers and post-polymerization halogenation strategies, there remains a clear gap in studies that investigate the one-step synthesis of pendant bromoacrylate polymers and their systematic transformation into multifunctional polymer platforms. In this work, we report the synthesis of a novel bromoacrylate monomer derived from cyclohexene *via* a bromonium-mediated acrylation reaction using NBS and excess acrylic acid. This monomer was subsequently polymerized under various solvent conditions to elucidate the effect of the reaction medium on monomer conversion, molecular weight, and dispersity. The retained C–Br functionality was then exploited for two independent post-polymerization transformations: nucleophilic substitution with sodium azide to introduce azide groups suitable for click-chemistry reactions, and quaternization with DABCO to produce partially DABCO-functionalized polymers.

## 2. Experimental

### 2.1. Materials

Cyclohexene (Merck, >99.0%), acrylic acid (Sigma-Aldrich, 99%), *N*-bromosuccinimide (Sigma-Aldrich, 99%), diethyl ether (Honeywell, >99.8%), potassium iodide (Sigma-Aldrich, 99%), sodium thiosulfate pentahydrate (Merck, ≥99.5%), sodium hydroxide (Acros Organics), sodium azide (Acros Organics; also Sigma-Aldrich), anhydrous sodium sulfate (Merck), silica gel (Merck, 0.063–0.200 mm), hexane (Sigma-Aldrich), ethyl acetate (Sigma-Aldrich), *N*,*N*-dimethylformamide (DMF, Sigma-Aldrich), methanol (Merck, 99.8%), and 1,4-diazabicyclo[2.2.2]octane (DABCO, Huntsman) were used as received. Azobisisobutyronitrile (AIBN, Sigma-Aldrich) was purified by recrystallization from methanol prior to use.

### 2.2. Instrumentation

^1^H NMR (500 MHz) and ^13^C NMR (125 MHz) spectra were recorded on a Bruker Avance III 500 MHz spectrometer at ambient temperature. All samples were dissolved in CDCl_3_; chemical shifts are reported in ppm relative to the residual solvent signal (*δ*^1^H = 7.26 ppm, *δ*^13^C = 77.0 ppm).

FTIR spectra were acquired on a PerkinElmer Spectrum One spectrometer over the range 400–4000 cm^−1^.

Molecular weight distributions were determined by gel permeation chromatography (GPC) at room temperature using an Agilent 1100 pump, an Agilent 1100 S refractive-index detector, and three Zorbax PSM columns in series (60 S, 300 S, and 1000 S; 6.2 × 250 mm; 5 µm particle size). THF was used as the mobile phase at a flow rate of 0.3 mL min^−1^. Number-average (*M*_n_) and weight-average (*M*_w_) molecular weights were determined relative to narrow polystyrene standards and the GPC solutions were filtered before injection.

Differential scanning calorimetry (DSC) measurements were conducted on a NETZSCH DSC 3500 Sirius instrument using aluminum Concavus pans with pierced lids under a nitrogen purge of 20 mL min^−1^. Samples of 3–9 mg were subjected to two complete heating – cooling cycles between 25 °C and 200 °C at 5 K min^−1^, with brief isothermal holds (∼1 min) at the temperature limits. Glass transition temperatures (*T*_g_) were extracted from the second-heating scan by the linear-intercept construction on baseline-corrected thermograms (NETZSCH Proteus software), plotted with endotherm up.

GC-MS analyses were performed on a PerkinElmer Clarus 500 GC coupled to a Clarus 500 S mass spectrometer operating in electron-ionization (EI) mode at 70 eV. Separations were carried out on an Elite-5MS capillary column (30 m × 0.25 mm i.d., 0.25 µm film) using helium (99.999%) as carrier gas at 1.0 mL min^−1^. Injector and ion-source temperatures were 250 °C and 230 °C, respectively; spectra were collected in full-scan mode over *m*/*z* 50–500.

Thermogravimetric analysis (TGA) was performed on a PerkinElmer Diamond TG/DTA system (Seiko Instruments SII Exstar 6300 module). Samples of 5–8 mg in platinum crucibles were heated from ambient temperature to 800 °C at 10 °C min^−1^ under flowing nitrogen (20–50 mL min^−1^). The onset degradation temperature (*T*_d_,_5%_), the temperature corresponding to 10% mass loss (*T*_d_,_10%_), and the temperature of maximum decomposition rate (*T*_max_) were extracted from derivative thermogravimetric (DTG) curves. Char residue was quantified at 700 °C. All measurements were performed at least in duplicate; representative curves are reported.

### 2.3. Synthetic procedures

#### 2.3.1. Monomer synthesis

The synthesis of 2-bromocyclohexyl acrylate was carried out following a previously reported procedure.^22^ Cyclohexene (1.15 g, 0.014 mol) and 150 mL of acrylic acid were placed in a round-bottomed flask and stirred for 10 minutes under a nitrogen atmosphere at room temperature. *N*-Bromosuccinimide (4.02 g, 0.022 mol) was then added and stirring was continued for a further 10 minutes. Once a clear solution had formed, the reaction mixture was allowed to stand in the dark at room temperature for 48 hours, affording a dark-yellow product with a characteristic clove-like aroma. Extraction was carried out using 100 mL of water and 150 mL of diethyl ether, in conjunction with aqueous solutions of KI (5 wt%, 100 mL) and Na_2_S_2_O_3_·5H_2_O (5 wt%, 100 mL) to remove excess NBS. Subsequently, 50 mL of 5% aqueous NaOH was applied to remove residual acrylic acid. Caution: The solution becomes hot upon addition of NaOH; it should therefore be introduced slowly and carefully under controlled conditions to avoid rapid heating or splashing. Finally, Na_2_SO_4_ was added to the organic phase, which was filtered and concentrated under vacuum to give a pale-yellow crude product. Purification by column chromatography on silica gel (hexane : ethyl acetate 7 : 2, v/v) afforded the product at *R*_f_ = 0.8 by TLC. After collection of the pure fractions, Na_2_SO_4_ was added and filtered, and the solvent was removed under vacuum. A light-yellow product was obtained in 95% isolated yield.

^1^H NMR (CDCl_3_, 500 MHz) *δ* 6.37 (d, 1H), 6.07 (dd, 1H), 5.79 (d, 1H), 4.90 (m, 1H), 3.95 (m, 1H), 2.26 (m, 1H), 2.11 (m, 1H), 1.86–1.75 (m, 2H), 1.73–1.60 (m, 2H), 1.42–1.21 (m, 3H). ^13^C NMR (CDCl_3_, 125 MHz) *δ* 165, 130, 128, 79, 53, 35, 30, 28, 23. FTIR: *ν̃* (C=O) 1722 cm^−1^, (C=C) 1636 cm^−1^, (C–O–C) 1182 cm^−1^, (C–Br) 695 cm^−1^.

#### 2.3.2. Polymer synthesis

Polymerizations were carried out under various solvent and reaction conditions, as summarized in Table 1. A representative procedure for the synthesis of P2 is as follows: Monomer (0.25 g, 1.077 × 10^−3^ mol) was dissolved in DMF (2 mL) in a septum-capped vial and stirred at 400 rpm for 5 min under nitrogen at room temperature. AIBN (0.0035 g, 2.154 × 10^−5^ mol) was dissolved in DMF (1 mL) in a separate vial and added to the monomer solution in a single portion. The mixture was heated to 85 °C and maintained at this temperature for 6 h under a static nitrogen atmosphere. After cooling to room temperature, the reaction mixture was precipitated into excess cold methanol and the resulting solid was collected by filtration and dried under vacuum to afford a white polymer in 34% yield. The same solution polymerization procedure was used for P3, differing only in the solvent employed 1,4-dioxane.

**Table 1:** Polymerization conditions and isolated yields of polymers P1–P3

No	Monomer (g)	Initiator AIBN (g)	Solvent	Time (hour)	Temp. (°C)	Isolated yield (%)
P1	0.25	0.0035	Bulk (neat)	6	85	65
P2	0.25	0.0035	DMF	6	85	34
P3	0.25	0.0035	Dioxane	6	85	49

Bulk polymerization (P1). Monomer (0.25 g, 1.077 × 10^−3^ mol) was placed directly into a septum-capped vial without solvent. AIBN (0.0035 g, 2.154 × 10^−5^ mol) was added directly to the monomer and the mixture was stirred briefly until homogeneous under nitrogen. The vial was heated at 85 °C for 6 h. After cooling, the crude polymer was dissolved in a minimum amount of 1,4-dioxane and precipitated into excess cold methanol. The polymer was collected by filtration and dried under vacuum to afford P1 in 65% isolated yield.

#### 2.3.3. Silver nitrate test

The presence of free bromide ions was assessed by adding 2 mL of 1 M AgNO_3_ solution to 5 mL of the methanolic polymer solution. Formation of a cloudy, turbid suspension indicated the presence of bromide. Appropriate positive controls (HBr, Br_2_, 1,1,2,2-tetrabromoethane) and negative controls (ethanol, methanol, THF, toluene, distilled water, and tap water) were run in parallel. This test was employed to assess whether bromine had been cleaved from the polymer during free-radical polymerization.

#### 2.3.4. Quaternization with DABCO

Polymer P1 (100 mg) was dissolved in DMF (1 mL) in an amber vial equipped with a septum cap, and DABCO (0.010 g) was added. Quaternization was carried out at 140 °C for 4 hours, during which a precipitate formed. The mixture was cooled to room temperature and washed with excess diethyl ether to remove unreacted DABCO and residual solvent. The precipitate was dried under vacuum at room temperature to constant mass.

#### 2.3.5. Azide substitution

A mixture of polymer P1 (100 mg) and sodium azide (50 mg) in DMF (5 mL) was stirred under a nitrogen atmosphere at 70 °C for 48 hours. After cooling to room temperature, the reaction mixture was diluted with ethyl acetate (40 mL) and extracted with water (3 × 20 mL). The organic layer was collected, dried, and the solvent was removed under reduced pressure. The obtained solid was dried under vacuum to constant mass.

## 3. Results and discussion

### 3.1. Synthesis and characterization of the bromoacrylate functional cyclohexane monomer (M1)

**Scheme 1:**
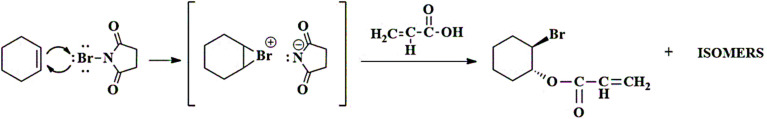
Synthesis of cyclohexane derivative of bromofunctional acrylate monomer.

The bromoacrylate functional cyclohexane monomer (M1) was synthesized from cyclohexene, acrylic acid, and NBS. It is well established that the bromination of an alkene in a nucleophilic solvent can lead to incorporation of the solvent as a nucleophile.^23^ To achieve a high yield of acrylic acid incorporation, it is necessary to maintain a low bromide ion concentration in order to minimize competitive dibromide formation. Scheme 1 illustrates the proposed mechanism of the bromoacrylation of cyclohexene. The reaction proceeds *via* heterolytic cleavage of the N–Br bond in NBS upon activation of the alkene π-system, generating a cyclic bromonium ion and a succinimide anion. The succinimide anion is subsequently protonated by the acidic reaction medium, producing succinimide as a weak nucleophile. Concurrently, acrylic acid—present in large excess and acting as both solvent and reagent—attacks the bromonium ion, resulting in regioselective introduction of the acrylate functionality. The use of excess acrylic acid is therefore essential for efficient solvent incorporation.

1,2-Disubstituted cyclohexanes can adopt multiple conformations and exist as stereoisomers depending on the nature and orientation of the substituents. The presence of two contiguous chiral centers gives rise to four possible stereoisomers (Fig. S1). In this work, only a simplified structural representation of the monomer is presented; however, a range of conformational and stereochemical possibilities must be considered when the chair conformation is taken into account. Among these, the *trans*-diequatorial 1,2-substitution pattern is predicted to be the most thermodynamically favorable. The geometries of the bromoacrylate stereoisomers were optimized using density functional theory (DFT) as implemented in Gaussian 09, employing the B3LYP hybrid functional—which incorporates Hartree–Fock and Becke exchange with the Lee–Yang–Parr correlation functional—together with the 6-31G(d) basis set. The calculations confirm that the *trans* conformer is the most stable form, exhibiting a lower total energy than the corresponding *cis* isomer (Fig. S2).

**Fig. 1:**
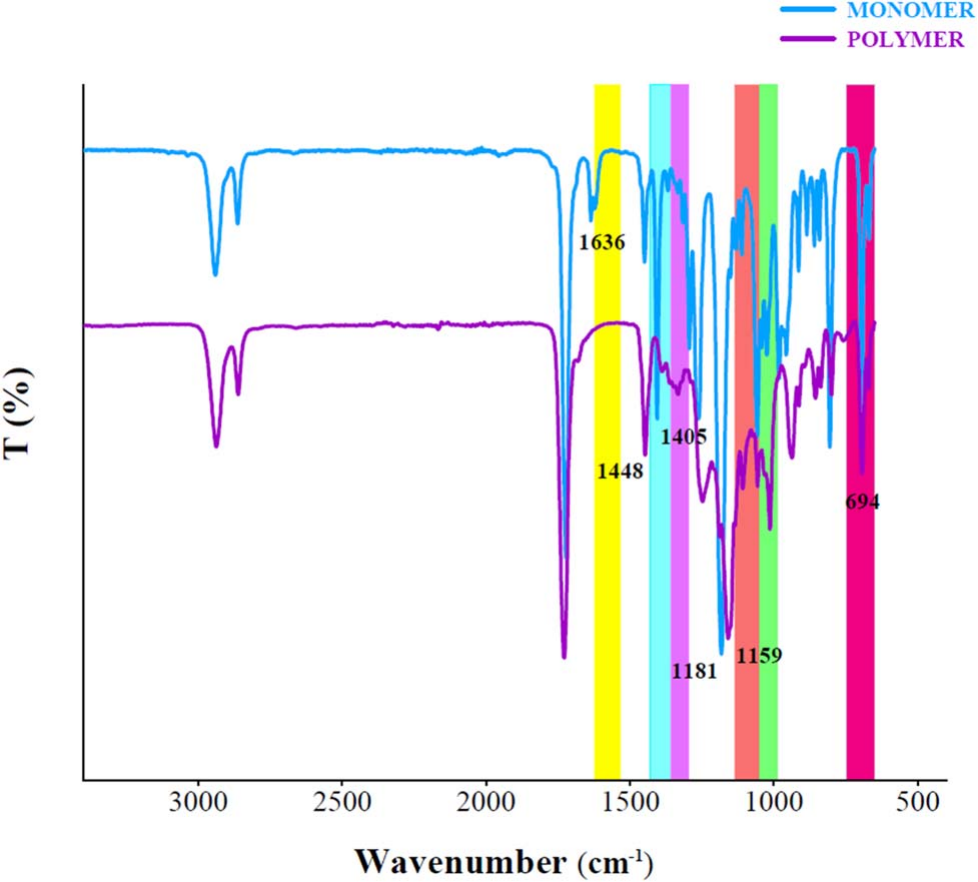
FTIR spectra of M1 and P1.

Monomer M1 was characterized by FTIR, ^1^H NMR, and ^13^C NMR spectroscopy. The FTIR spectrum is shown in Fig. 1. Characteristic bands associated with the acrylate functionality are clearly observed: carbonyl (C=O) stretching at 1729 cm^−1^, C=C stretching of the vinyl group at ∼1636 cm^−1^, and C–O–C asymmetric stretching at 1182 cm^−1^. Additional bands at 1406 cm^−1^ (C–H bending) and 695 cm^−1^ (C–Br stretching) confirm the bifunctional character of the monomer and the successful incorporation of bromine.^24^

**Fig. 2:**
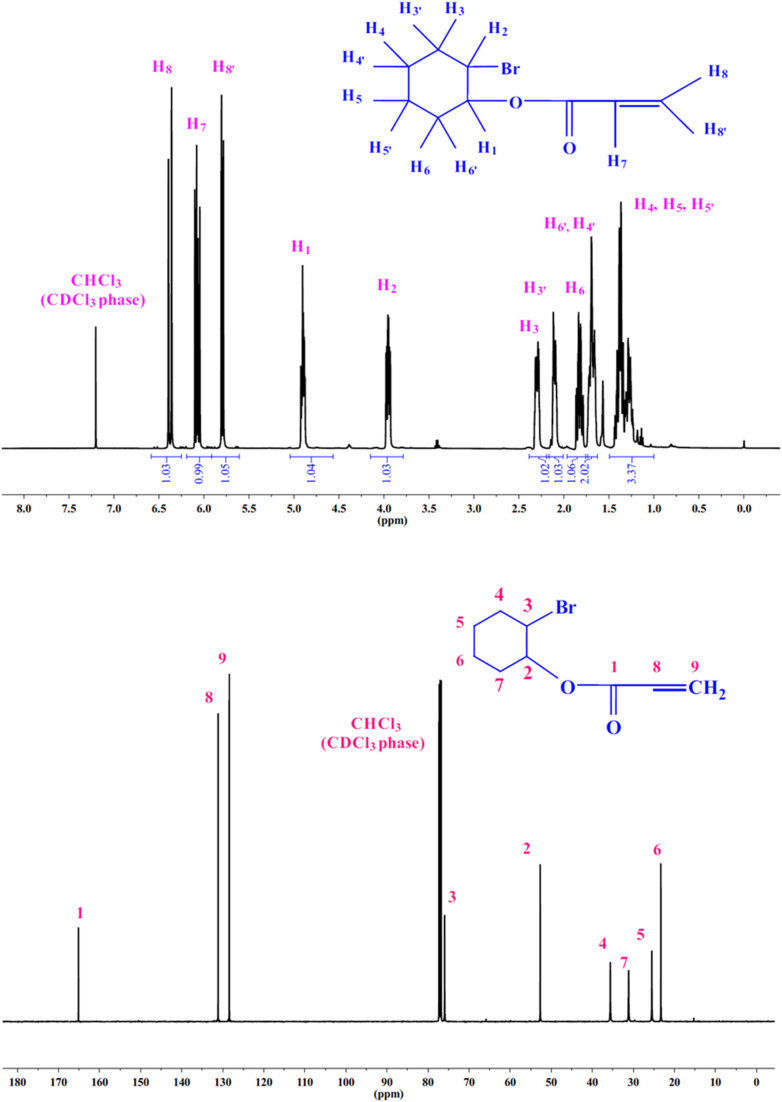
^1^H NMR (up) and ^13^C NMR (down) spectrum spectra of bromoacrylate functional monomer (M1).

The ^1^H NMR spectrum of M1 is shown in Fig. 2. The methine proton geminal to bromine (–CHBr–) resonates at 3.95 ppm, and the methine proton of the acyloxy-bearing carbon (–CHOCOCH=CH_2_) appears at 4.90 ppm. The three vinyl protons of the acrylate group give rise to a characteristic pattern at 6.37 (d), 6.07 (dd), and 5.79 (d) ppm. The remaining cyclohexane ring protons appear as complex multiplets between 2.43 and 1.19 ppm. The presence of multiple stereoisomers arising from the relative *cis*/*trans* orientation of the bromine and acrylate substituents on the cyclohexane ring is clearly reflected in the spectrum. Stereochemical assignment of the predominant isomer is based on analysis of the vicinal coupling constants (^3^*J*) between the protons on the substituted ring carbons. In accordance with the Karplus relationship, axial–axial (*trans*-diaxial) couplings exhibit larger values (typically 8–12 Hz), whereas axial – equatorial (*cis*) couplings are smaller (2–5 Hz). The dominant isomer displays the larger vicinal coupling constants, consistent with a *trans* configuration in which both substituents preferentially adopt equatorial positions in the most stable chair conformer.

The ^13^C NMR spectrum of M1 is also shown in Fig. 2. The carbonyl resonance appears at 165 ppm. Signals at 130 and 128 ppm are assigned to the methylene (–CH_2_) carbon adjacent to the acrylate group and the methine (–CH) carbon bonded to the acrylate oxygen, respectively. The oxymethine carbon (–CHOC=O–) appears at 53 ppm, and the carbon bearing the bromine substituent is observed at 79 ppm.

GC-MS analysis of M1 revealed a prominent fragment ion cluster at *m*/*z* 159.9/161.1, rather than at the calculated molecular mass of 233.1 g mol^−1^. The characteristic ≈1 : 1 isotope pattern is diagnostic for the presence of a single bromine atom (^79^Br/^81^Br) in the fragment ion (Fig. S3). The absence of the intact molecular ion [M]^+^ and the observation of a lower-mass fragment are attributed to the well-known propensity of secondary alkyl bromides and brominated acrylates to undergo facile C–Br bond cleavage or HBr elimination under 70 eV electron-ionization conditions, generating stable carbocationic species in the *m*/*z* 160 region. The observed fragmentation behavior is therefore fully consistent with the proposed structure.

### 3.2. Polymer synthesis

**Scheme 2:**
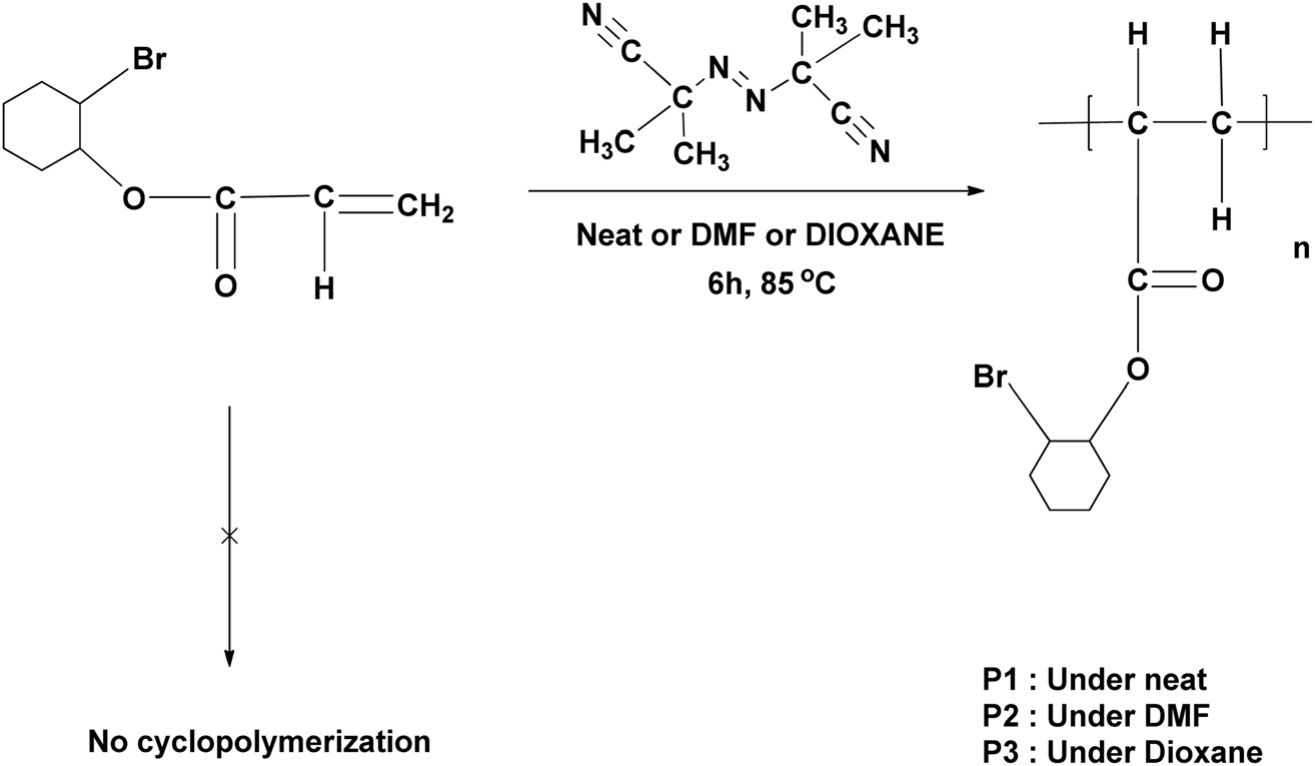
Polymerization of bromoacrylate functional cyclohexane monomer (M1).

Scheme 2 presents the polymerization route. Initially, various polymerization conditions, including temperature, solvent, and reaction time, were investigated to determine whether cyclopolymerization could occur through the formation of thermodynamically favorable five- or six-membered rings following cleavage of the bromine substituent. To clarify this point, a proposed mechanistic scheme (Scheme S1) has been included in the SI to illustrate the possible intramolecular cyclization pathway. Our initial hypothesis was that, following radical addition to the vinyl group, the pendant brominated side chain could undergo intramolecular nucleophilic substitution or radical cyclization to generate thermodynamically favorable five- or six-membered ring structures.

However, the experimental results do not provide convincing evidence that such cyclization occurs to a significant extent. In particular, the ^1^H NMR spectra show that the CH–Br proton signal is largely retained after polymerization, indicating that cleavage of the C–Br bond and subsequent ring formation are not major pathways under the polymerization conditions employed.

Three distinct reaction conditions were investigated: bulk polymerization (P1), and solution polymerization in DMF (P2) and 1,4-dioxane (P3), all conducted at 85 °C with AIBN as the radical initiator. FTIR and ^1^H NMR analyses confirmed successful polymerization while demonstrating that the brominated side chains remained largely intact. Analysis of the methanol filtrate using the AgNO_3_ test indicated that only trace amounts of bromide were released during polymerization, further supporting the conclusion that bromine cleavage was minimal under the reaction conditions employed.

Representative characterization data are presented for P1. The FTIR spectrum in Fig. 1 confirms complete consumption of the acrylate double bond, as evidenced by the disappearance of the C=C stretching band at 1636 cm^−1^.^25,26^ The ester carbonyl band at 1727 cm^−1^ and the C–O–C band at 1182 cm^−1^ remain clearly visible. Importantly, the persistence of the C–Br stretching band at 694 cm^−1^ in the polymer spectrum demonstrates that bromine is not completely cleaved from the structure during polymerization. All three polymers exhibited essentially identical FTIR spectra.

**Fig. 3:**
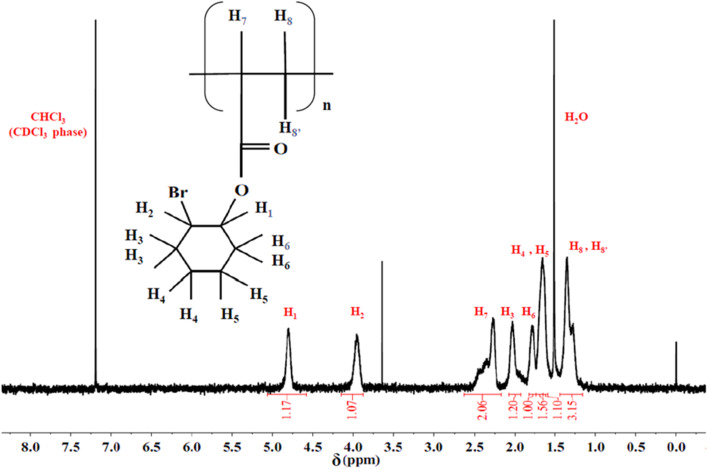
^1^H NMR spectrum of P1.

Successful polymerization was further confirmed by ^1^H NMR spectroscopy. In the spectrum of P1 (Fig. 3), the characteristic vinyl proton signals at 5.79, 6.12, and 6.37 ppm are absent, and new aliphatic backbone resonances appear at *δ* 1.35 and 2.35 ppm corresponding to the main-chain –CH_2_– and –CH– protons. The cyclohexane ring protons are observed in the range *δ* 2.03–2.52 ppm. The ^1^H NMR spectra of P2 and P3 are provided in the SI (Fig. S4 and S5).

In the ^1^H NMR spectrum of the polymer, the –CH–Br resonance is observed at 3.9 ppm, coinciding with its position in the monomer spectrum. This indicates that the bromine atom is not cleaved quantitatively during polymerization. During free-radical polymerization, bromine can participate in cleavage reactions—particularly through radical-induced abstraction—and may thereby influence chain kinetics, molecular weight, and the properties of the resulting polymer.^27^ Qualitative AgNO_3_ testing was performed to assess bromine retention in the polymer structure. The AgNO_3_ test applied to the precipitating methanol phase showed only slight turbidity, indicating that a minor quantity of bromine is released during polymerization. This cleavage is quantitatively small, as supported by ^1^H NMR integration: the –CH–Br and –CH–O(C=O) signals, which should integrate equally as single protons per repeat unit, show a ∼5% deficit in the –CH–Br integral, indicating that only a small fraction of bromine is lost during the polymerization process.

The molecular weights of the polymers were determined by GPC; the results are summarized in Table 2. The key parameters evaluated are the number-average molecular weight (*M*_n_), weight-average molecular weight (*M*_w_), and dispersity index (*Đ* = *M*_w_/*M*_n_).

**Table 2:** Molecular weight by GPC analysis of polymers

Sample	*M*_n_ (g mol^−1^)	*M*_w_ (g mol^−1^)	*Đ* (*M*_w_/*M*_n_)
P1	195 000	1 168 000	5.98
P2	7550	10 060	1.33
P3	8840	12 030	1.36

P1, prepared under bulk (neat) conditions, exhibited a very high molecular weight. However, the exceptionally broad molecular weight distribution (*Đ* = 5.98) is characteristic of an uncontrolled radical polymerization process subject to significant chain-length heterogeneity, consistent with the onset of the Trommsdorff–Norrish (gel) effect at high monomer conversion.

In contrast, solution polymerizations in DMF (P2) and 1,4-dioxane (P3) afforded lower molecular weights together with narrower apparent dispersities (*Đ* = 1.33 and 1.36, respectively). Although these values were reproducible, they are relatively low for conventional AIBN-initiated free-radical polymerization. Their origin remains uncertain and may reflect factors related to the polymer architecture, differences in hydrodynamic behavior during GPC analysis, or other experimental variables. Further investigation using orthogonal molecular-weight characterization techniques, such as GPC-MALS or MALDI-TOF mass spectrometry, would be valuable to clarify this observation. The lower molecular weights observed for P2 and P3 may be associated with solvent effects, including chain-transfer reactions or enhanced termination events that limit chain growth. The retention time shifts observed in the GPC chromatograms (Fig. S6) are consistent with these trends, with P1 eluting earlier than P2 and P3 owing to its larger apparent hydrodynamic volume.

It should be noted that the molecular weights were determined by GPC using linear polystyrene standards; therefore, the reported values represent relative rather than absolute molecular weights. Because the brominated polyacrylates synthesized in this study possess bulky pendant groups and may adopt solution conformations different from those of linear polystyrene, their hydrodynamic volumes may not directly correlate with those of the calibration standards. Consequently, deviations in the absolute molecular weight values are expected. Nevertheless, because all samples were analyzed under identical chromatographic conditions, the GPC data provide a reliable basis for comparing the relative influence of the polymerization medium on the apparent molecular-weight distribution of the polymers. Overall, the results demonstrate that the polymerization medium has a significant influence on the apparent molecular-weight characteristics of the resulting polymers. Bulk polymerization produced a polymer with a substantially higher apparent molecular weight and a broad molecular-weight distribution, whereas solution polymerization yielded polymers with lower apparent molecular weights and narrower apparent dispersities.

#### 3.2.1. Thermal analysis

##### 3.2.1.1. TGA analysis

**Fig. 4:**
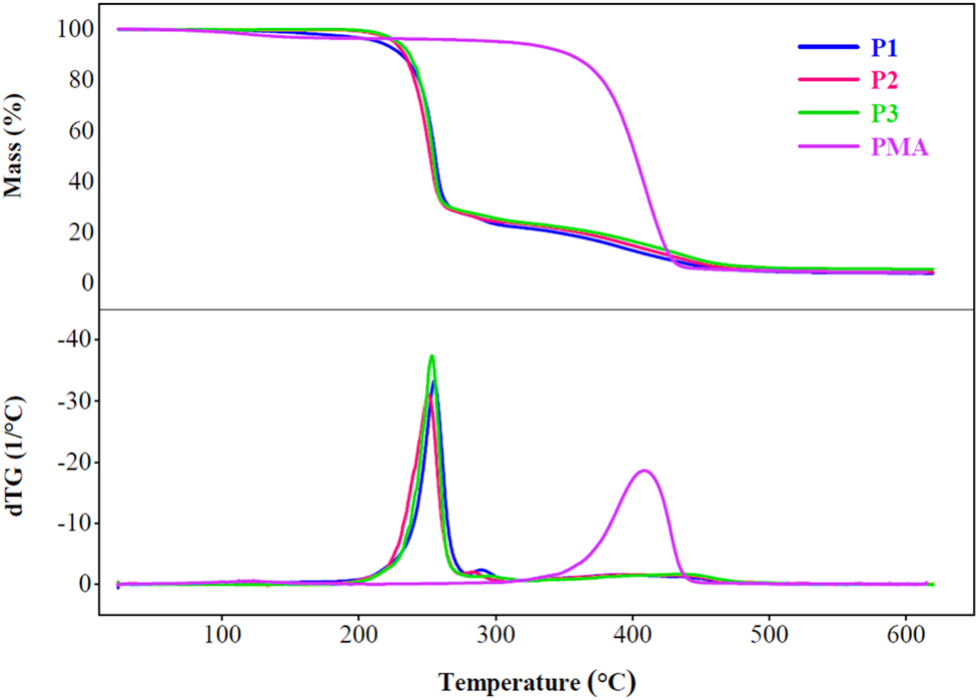
TGA/DTA graph of polymers, P1, P2, P3 and poly(methyl acrylate) (PMA).

Comparative thermogravimetric analysis (TGA) curves for P1, P2, P3, and poly(methyl acrylate) (PMA) are presented in Fig. 4. The corresponding thermal parameters, including *T*_d,5%_, *T*_d,10%_, *T*_max_, and char yield at 700 °C, are summarized in Table 3. A sharp mass loss was observed in all three polymers in the range 250–400 °C.^32–34^ The brominated polymers P1–P3 exhibited similar degradation profiles, with *T*_d,5%_ values ranging from 168 to 175 °C and maximum degradation rates near 280 °C. In contrast, PMA displayed significantly higher thermal stability, with a *T*_d,5%_ of approximately 340 °C and a *T*_max_ of 413.9 °C. These results indicate that bromine incorporation does not increase the intrinsic thermal stability of the polymer; rather, the lower onset temperatures are attributed to cleavage of the relatively weak C–Br bond and subsequent dehydrohalogenation.

**Table 3:** Thermal degradation parameters of P1–P3 and PMA determined by thermogravimetric analysis

Sample	*T*_d,5%_ (°C)	*T*_d,10%_ (°C)	*T*_max_ (°C)	Char yield at 700 °C (wt%)
P1	175	275	∼280	4.0
P2	168	282	∼280	4.5
P3	171	280	∼280	5.8
PMA	340	407.5	413.9	4.46

The char yields of P1, P2, and P3 at 700 °C were 4.0, 4.5, and 5.8 wt%, respectively, which are comparable to that of PMA (4.46 wt%). Therefore, bromination did not significantly enhance char formation under nitrogen. Nevertheless, qualitative vertical burning tests showed that filter paper coated with P1 exhibited self-extinguishing behavior, whereas untreated filter paper and filter paper coated with the non-brominated polyacrylate burned completely. Please see the vertical burning test analysis below.

##### 3.2.1.2. DSC analysis

**Fig. 5:**
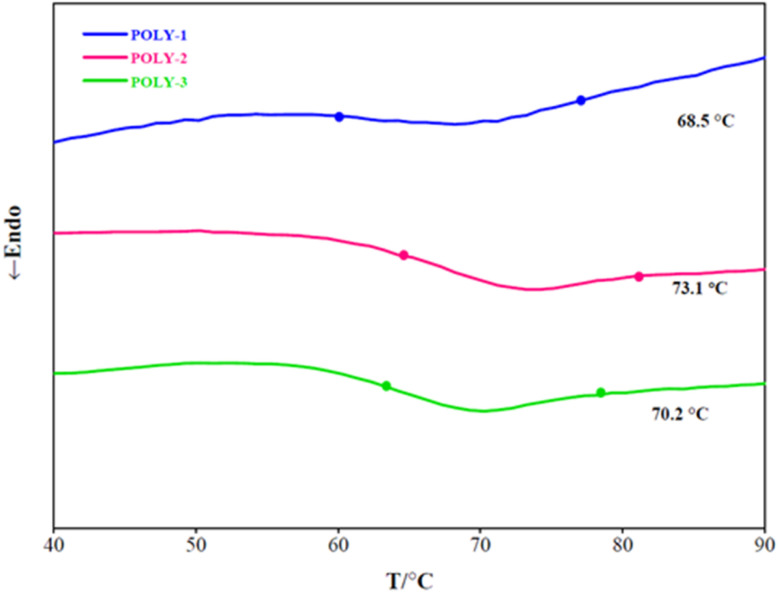
DSC graph of polymers.

Fig. 5 shows the DSC graph of polymers. Although the *T*_g_ differences within this series are modest, they provide meaningful insight into the interplay between chain structure and molecular-weight distribution. P2 exhibits the highest *T*_g_ (73.1 °C), which correlates strongly with its narrow dispersity (*Đ* = 1.33) and relatively uniform molar-mass distribution (Table 2). A lower proportion of short-chain species reduces the plasticization effect inherent to chain-end enrichment, enabling more efficient chain packing and stronger cohesive intermolecular interactions. These characteristics collectively suppress segmental mobility, resulting in an elevated *T*_g_. In contrast, P1 displays the lowest *T*_g_ (68.5 °C) despite possessing the highest number-average molecular weight. This apparent paradox is readily rationalized by its very broad dispersity (*Đ* = 5.98), which implies the presence of a substantial low-molecular-weight tail. These shorter chains act as an internal plasticizer, increasing free volume and enhancing chain mobility to an extent that more than offsets the *T*_g_ elevation expected from the high-molecular-weight fraction. P3 exhibits an intermediate *T*_g_ of ∼70.0 °C, consistent with its moderate molecular weight and dispersity (*Đ* = 1.36), reflecting a balance between chain mobility and cohesive intermolecular interactions.

Overall, the *T*_g_ trend of P2 > P3 > P1 demonstrates that segmental mobility is progressively restricted from P1 to P2. Taken together, the DSC and GPC data confirm that chain-length uniformity and molecular-level packing interactions—rather than absolute molecular weight alone—are the dominant factors governing thermal relaxation behavior in these bromo-functional acrylate polymers.

#### 3.2.2. Vertical burn test

The incorporation of bromine into the polymer structure is expected to impart flame-retardant properties due to the well-established radical-trapping mechanism of brominated compounds in the gas phase. To assess the potential self-extinguishing behavior of the synthesized polymers, a simple vertical burning test was performed using standard laboratory filter paper coated with either a non-brominated polyacrylate homopolymer or the brominated polymer P1.

The coating procedure involved immersing the filter paper in a chloroform solution of each polymer (0.2 g mL^−1^) for 3 min, followed by solvent removal under vacuum. As a control, untreated filter paper was subjected to the same test and burned completely within approximately 7 s, leaving no visible residue (Fig. S7a). Filter paper coated with the non-brominated polyacrylate homopolymer exhibited similar behavior (Fig. S7b), indicating that the polyacrylate matrix itself did not provide significant flame resistance.

In contrast, filter paper coated with polymer P1 ignited readily but self-extinguished within approximately 6 s after removal of the ignition source, leaving behind a charred residue that resisted further combustion (Fig. S7c). This behavior demonstrates the flame-retardant effect imparted by the brominated side groups.

The flame-retardant action of brominated polymers is generally attributed to the release of bromine radicals (Br˙) and hydrogen bromide during thermal decomposition. These species efficiently scavenge the highly reactive hydrogen (H˙) and hydroxyl (OH˙) radicals that propagate combustion, thereby interrupting the gas-phase radical chain reactions responsible for flame propagation.^28^ A comprehensive overview of commercially used BFRs, their applications, regional use patterns, and possible release pathways has been reported in the literature.^29–31^

Although the simple burning test confirmed the self-extinguishing behavior of polymer P1, an initial flare and a short period of sustained burning were still observed. These results indicate that bromination improves fire resistance; however, further studies using standardized flammability tests, such as cone calorimetry, UL-94 vertical burning, and limiting oxygen index (LOI) measurements, are required to quantitatively assess and optimize the flame-retardant performance of these materials.

### 3.3. Functionalization of the bromoacrylate polymer

#### 3.3.1. Quaternization with DABCO

**Scheme 3:**
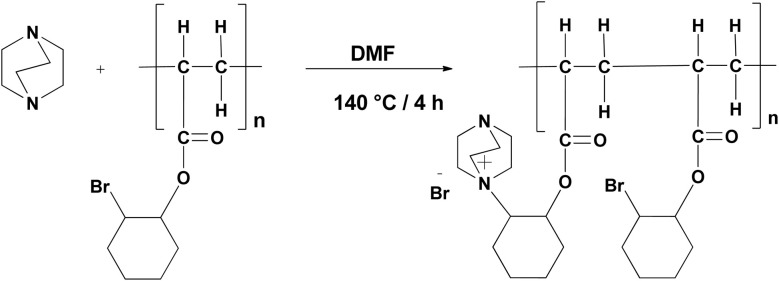
Quaternization of the polymer with DABCO.

In this section, we demonstrate one of the principal applications of the synthesized polymers as cationic building blocks. DABCO was reacted with the pendant alkyl bromide groups *via* S_N_2-type nucleophilic substitution. The resulting product is a quaternary ammonium salt in which the DABCO cage is attached to the polymer side chain through a newly formed N–C bond, imparting a permanent positive charge to the repeat unit. The monoquaternization pathway is illustrated in Scheme 3. Comparative FTIR analysis of the parent polymer and the DABCO-quaternized product was presented in Fig. S8. After quaternization with DABCO, the characteristic ester carbonyl stretching of the polyacrylate backbone was retained at approximately 1730–1750 cm^−1^, indicating preservation of the ester functionality. The decrease in the C–Br stretching band in the 650–700 cm^−1^ region, together with the appearance/intensification of C–N stretching bands around 1050–1150 cm^−1^ supports the successful nucleophilic substitution of the brominated side groups and formation of DABCO-based quaternary ammonium moieties.

The ^13^C NMR spectrum of the quaternized product provides further structural support (Fig. S9): a carbonyl signal at 163 ppm; the ring carbon bonded to oxygen at 63 ppm and the carbon bonded to nitrogen at 58 ppm; bridging carbons of the DABCO cage at 52 and 53 ppm; methylene carbons at 43 and 44 ppm; and additional aliphatic signals at 37, 34, 31, and 15 ppm.

During the quaternization study, several reaction conditions were investigated to maximize the reaction between the brominated polymer and DABCO. Under highly concentrated conditions, rapid gelation was observed, likely due to intermolecular crosslinking. The resulting materials became insoluble and therefore could not be characterized by solution-state NMR or GPC. To enable structural characterization, subsequent experiments were conducted under more dilute conditions, affording soluble products that were suitable for analysis, although these samples likely exhibited a lower degree of quaternization.

##### 3.3.1.1. Conductivity measurement

After quaternization, the product was precipitated into diethyl ether, collected by filtration, and dried under vacuum. Thin films for conductivity measurements were prepared by cold pressing the polymer powder using a hydraulic press at 2 MPa for 15 s, yielding discs with a diameter of 1 cm and a thickness of approximately 0.05 mm. The ionic conductivity of the resulting partially quaternized DABCO-functionalized polymer was measured to be 6.00 × 10^−8^ S cm^−1^.

The relatively low value of conductivity does not support describing the material as highly conductive. Accordingly, the product is more appropriately referred to as a partially quaternized DABCO-functionalized polymer rather than a conductive polyelectrolyte. The low conductivity is likely attributable to the limited degree of quaternization and the heterogeneous nature of the cold-pressed films used for conductivity measurements.

#### 3.3.2. Polymer-azide reaction

**Scheme 4:**
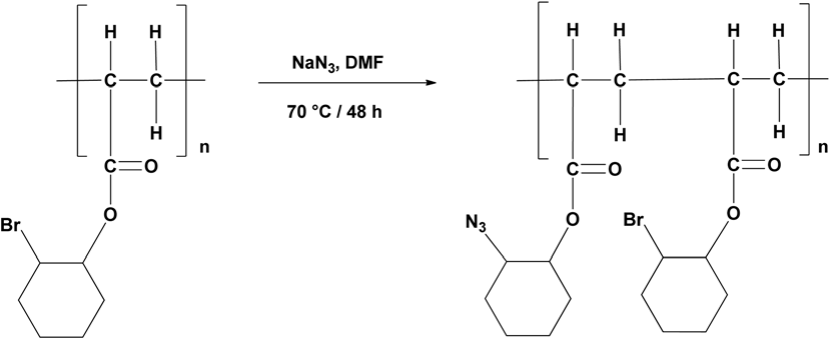
Post-functionalization of polymer *via* azide substitution.

In the azide functionalization study, experiments were conducted to evaluate whether the synthesized 2-bromoacrylate polymer could be converted into an azide-functionalized polymer suitable for copper-catalyzed or thermal azide–alkyne cycloaddition (“click”) reactions. The polymer was reacted with sodium azide (NaN_3_) in various solvents using different reagent ratios, temperatures, and reaction times to identify suitable reaction conditions. In a representative experiment, the bromoacrylate polymer was treated with sodium azide in DMF at 70 °C for 48 h (Scheme 4). After completion of the reaction, the product was isolated by extraction with water and ethyl acetate to remove inorganic salts and residual reagents. The ethyl acetate phase was concentrated under reduced pressure to a small volume and then precipitated twice into cold methanol. The resulting solid was collected by filtration and dried under vacuum.

The results indicated that azide substitution proceeded only partially under the conditions investigated. The incomplete conversion is likely attributable to a combination of factors, including steric hindrance around the secondary bromide center, restricted segmental mobility of the polymer chains, and limited accessibility of reactive sites within the polymer matrix.

FTIR analysis of the product (Fig. S10) shows C–H stretching vibrations of the cyclohexane ring at 2937–2861 cm^−1^. A band of weak intensity at 2100 cm^−1^, assigned to the asymmetric stretching vibration of the azide (N_3_) group, is observed and is indicative of partial azide incorporation. The ester carbonyl band of the polymer persists at 1721 cm^−1^. Aliphatic CH_2_/CH_3_ bending vibrations appear at 1447–1379 cm^−1^. The bands in the 1258–1013 cm^−1^ region are assigned to C–O–C stretching, C–N, and C–O vibrations characteristic of the ester linkage. The band at 693 cm^−1^, corresponding to C–Br stretching, remains clearly present, indicating that a significant fraction of bromine atoms have not undergone substitution completely. In the ^1^H NMR spectrum of the azide-functionalized polymer (Fig. S11), the integrity of the backbone is preserved while the azide conversion is evidently incomplete. The signal at 5.1–4.9 ppm (–CH–O–, H1) confirms retention of the ester linkage. The –CH–Br protons appear at 3.9–3.8 ppm, and their persistence confirms that bromine-bearing repeat units remain and that the azide substitution is incomplete. A weak signal at 3.2 ppm is tentatively assigned to –CH–N_3_, providing direct spectroscopic evidence for azide incorporation. The complex multiplet region at 2.2–1.2 ppm encompasses the aliphatic cyclohexane ring protons.

The primary objective of this experiment was to demonstrate the feasibility of post-polymerization modification of the brominated polymer backbone. We are currently conducting a more comprehensive study focused on azide-functionalized polymers and their subsequent copper-catalyzed azide–alkyne cycloaddition (CuAAC) reactions with BODIPY derivatives. In that work, the optimization of azidation conditions and quantitative evaluation of substitution efficiency will be examined in greater detail.

## 4. Conclusion

This study reports the successful synthesis and comprehensive characterization of a series of bromoacrylate-based polymers and their corresponding functionalized derivatives. The monomer was efficiently prepared in high yield *via* bromoacrylation of cyclohexene with acrylic acid and NBS, and subsequent free-radical polymerization under three distinct reaction conditions produced polymers with well-retained bromine functionalities. Polymerization conditions were shown to exert a strong influence on molecular weight and dispersity. Thermal analysis showed that bromine incorporation did not enhance intrinsic thermal stability or significantly increase char yield. However, qualitative vertical burning tests demonstrated self-extinguishing behavior, suggesting that the flame-retardant effect arises predominantly from gas-phase radical scavenging rather than enhanced char formation. Quaternization with DABCO further expanded the functional scope of these materials, demonstrating the feasibility of post-polymerization DABCO functionalization. These findings collectively demonstrate that brominated acrylate polymers derived from cyclohexene serve as versatile platform materials for multifunctional applications. The incorporation of bromine-containing side groups, together with subsequent transformation into cationic or azide-functional polymers, imparts significant multifunctionality to these materials. Future work will focus on evaluating the performance of these materials in antimicrobial, ion-transport, and click-chemistry-based applications.

## Author contributions

Ülfet Akgün: formal analysis, writing – review & editing, writing – original draft, validation, methodology, investigation, Arzu Hatipoğlu: formal analysis, conceptualization, methodology, Tarık Eren: resources, project administration, writing – review & editing, validation, supervision, methodology, formal analysis, data curation, conceptualization.

## Conflicts of interest

The authors declare no conflict of interest.

## Supplementary Material

RA-OLF-D6RA02373H-s001

## Data Availability

The data supporting this article have been included as part of the supplementary information (SI). Supplementary information: FTIR, NMR, DSC spectra and further experimental details. See DOI: https://doi.org/10.1039/d6ra02373h.

## References

[cit1] Schneiderman D. K., Hillmyer M. A. (2017). 50th Anniversary Perspective: There Is a Great Future in Sustainable Polymers. Macromolecules.

[cit2] Kang T. W., Park K., Kim M. S. (2026). Advances in Stimuli-Responsive Polymers for Biomedical and Environmental Applications. Mater. Sci. Eng. R Rep..

[cit3] Kumar A., Thakur V. K., Nezhad H. Y., Lee K.-S. (2024). Prospects of Sustainable Polymers. Sci. Rep..

[cit4] Wang B., Zhan L., Guan C. (2025). Advanced Polymer Composites and Applications. Polymers.

[cit5] Kamran F., Afshar H., Shahi F. (2025). Recent Advances and Applications of Sustainable and Recyclable Polymers. Polym. Eng. Sci..

[cit6] de Boer J., Leonards P. E. G., Zennegg M. (2024). The European Regulatory Strategy for Flame Retardants. Chemosphere.

[cit7] Berger G., Soubhye J., Meyer F. (2015). Halogen Bonding in Polymer Science: From Crystal Engineering to Functional Supramolecular Polymers and Materials. Polym. Chem..

[cit8] Wang C. G., Chong A. M. L., Pan H. M., Sarkar J., Tay X. T., Goto A. (2020). Recent Development in Halogen-Bonding-Catalyzed Living Radical Polymerization. Polym. Chem..

[cit9] Kampes R., Zechel S., Hager M. D., Schubert U. S. (2021). Halogen Bonding in Polymer Science: Towards New Smart Materials. Chem. Sci..

[cit10] Kakuchi R. and Theato P., Post-Polymerization Modifications via Active Esters. in Functional Polymers by Post-Polymerization Modification, ed P. Theato and H.-A. Klok, 2012, 10.1002/9783527655427.ch2

[cit11] Parcheta M., Sobiesiak M. (2023). Preparation and Functionalization of Polymers with Antibacterial Properties—Review of the Recent Developments. Materials.

[cit12] Bromberg L., Pomerantz N., Schreuder-Gibson H., Hatton T. A. (2014). Degradation of Chemical Threats by Brominated Polymer Networks. Ind. Eng. Chem. Res..

[cit13] Blauer G., Shenblat M., Katchalsky A. (1959). Polymerization of Vinyl Bromide in Solution. J. Polym. Sci..

[cit14] Rusu M. C., Ichim I. C., Popa M., Rusu M. (2008). New Radiopaque Acrylic Bone Cement. II. Acrylic Bone Cement with Bromine-Containing Monomer. J. Mater. Sci.: Mater. Med..

[cit15] Mansour S. H., Asaad J. N., Abd-El-Messieh S. L. (2006). Synthesis and Characterization of Brominated Polyester Composites. J. Appl. Polym. Sci..

[cit16] Technique Puts Bromine in Polyesters, Chem. Eng. News, 1964, 42(9), pp. 56–57, 10.1021/cen-v042n009.p056

[cit17] Buzdugan E., Ghioca P., Badea E. G., Serban S., Stribeck N. (1997). Bromination of Some Styrene Diene Block Copolymers. Eur. Polym. J..

[cit18] Cao R., Zhao X., Zhao X., Wu X., Li X., Zhang L. (2019). Bromination Modification of Butyl Rubber and Its Structure, Properties, and Application. Ind. Eng. Chem. Res..

[cit19] Choothong N., Kosugi K., Yamamoto Y., Kawahara S. (2017). Characterization of Brominated Natural Rubber by Solution-State
2D NMR Spectroscopy. React. Funct. Polym..

[cit20] Zhou H., Chen Y., Plummer C. M., Huang H., Chen Y. (2017). Facile and Efficient Bromination of Hydroxyl-Containing Polymers to Synthesize Well-Defined Brominated Polymers. Polym. Chem..

[cit21] Mountaki S. A., Whitfield R., Parkatzidis K., Antonopoulou M.-N., Truong N. P., Anastasaki A. (2024). Chemical Recycling of Bromine-Terminated Polymers Synthesized by ATRP. RSC Appl. Polym..

[cit22] Eren T., Küsefoǧlu S. H. (2004). Synthesis and Characterization of Copolymers of Bromoacrylated Methyl Oleate. J. Appl. Polym. Sci..

[cit23] Djerassi C. (1948). Brominations with N-Bromosuccinimide and Related Compounds. The Wohl-Ziegler Reaction. Chem. Rev..

[cit24] Clive D. L. J., Zhang J., Subedi R., Bouétard V., Hiebert S., Ewanuk R. (2001). Preparation of α-(2,2-Diphenylhydrazino)Lactones and Related Compounds by Radical Cyclization: Use of Glyoxylic Acid Hydrazone Derivatives. J. Org. Chem..

[cit25] Nandiyanto A. B. D., Ragadhita R., Fiandini M. (2022). Interpretation of Fourier Transform Infrared Spectra (FTIR): A Practical Approach in the Polymer/Plastic Thermal Decomposition. Indones. J. Sci. Technol..

[cit26] Litvinov V. M. and De P. P., Spectroscopy of Rubbers and Rubbery Materials, Rapra Technology Limited, Shawbury, UK, 2002

[cit27] Hutchins-Crawford H. J., Ninjiaranai P., Derry M. J., Molloy R., Tighe B. J., Topham P. D. (2021). Bromoform-Assisted Aqueous Free Radical Polymerisation: A Simple, Inexpensive Route for the Preparation of Block Copolymers. Polym. Chem..

[cit28] Altarawneh M., Saeed A., Al-Harahsheh M., Dlugogorski B. Z. (2019). Thermal Decomposition of Brominated Flame Retardants (BFRs): Products and Mechanisms. Prog. Energy Combust. Sci..

[cit29] Alaee M., Arias P., Sjödin A., Bergman Å. (2003). An Overview of Commercially Used Brominated Flame Retardants, Their Applications, Their Use Patterns in Different Countries/Regions and Possible Modes of Release. Environ. Int..

[cit30] Koch C., Nachev M., Klein J., Köster D., Schmitz O. J., Schmidt T. C., Sures B. (2019). Degradation of the Polymeric Brominated Flame Retardant “Polymeric FR” by Heat and UV Exposure. Environ. Sci. Technol..

[cit31] Horrocks A. R. (2020). The Potential for Bio-Sustainable Organobromine-Containing Flame Retardant Formulations for Textile Applications-a Review. Polymers.

[cit32] Dhal P. K., Babu G. N., Chien J. C. W. (1987). Resist Polymers: Part VIII-Thermolysis of Bromine-Containing Acrylate Polymers. Polym. Degrad. Stab..

[cit33] Koca M., Kurt A., Kirilmis C., Aydogdu Y. (2012). Synthesis, Characterization, and Thermal Degradation of Novel Poly(2-(5-Bromo Benzofuran-2-Yl)-2-Oxoethyl Methacrylate). Polym. Eng. Sci..

[cit34] Grassie N., Johnston A., Scotney A. (1981). Thermal Degradation of Bromine-Containing Polymers. Part 2—Characteristics and Products of Degradation of Copolymers of 2-Bromoethyl Methacrylate and Methyl Acrylate. Polym. Degrad. Stab..

