# Supplementary material for: Synthesis and characterization of bromo-functional acrylate polymers
Source: RSC Adv. 2026 Jul 23. Online ahead of print. doi: 10.1039/d6ra02373h (PMC13392991; doi:10.1039/d6ra02373h)
Supplement: RA-OLF-D6RA02373H-s001 [file RA-OLF-D6RA02373H-s001.pdf]

Supplementary Information (SI) for RSC Advances.
This journal is © The Royal Society of Chemistry 2026

Supplementary Information

SYNTHESIS AND CHARACTERIZATION OF BROMO-FUNCTIONAL ACRYLATE POLYMERS

Ulfet Akgun [1], Arzu Hatipoglu [1], Tarik Eren [1,*]

[1] *Department of Chemistry, Faculty of Art and Science, Yildiz Technical University, Istanbul, 34220, Turkey*

***E - mail:** *teren@yildiz.edu.tr; erentari@gmail.com*

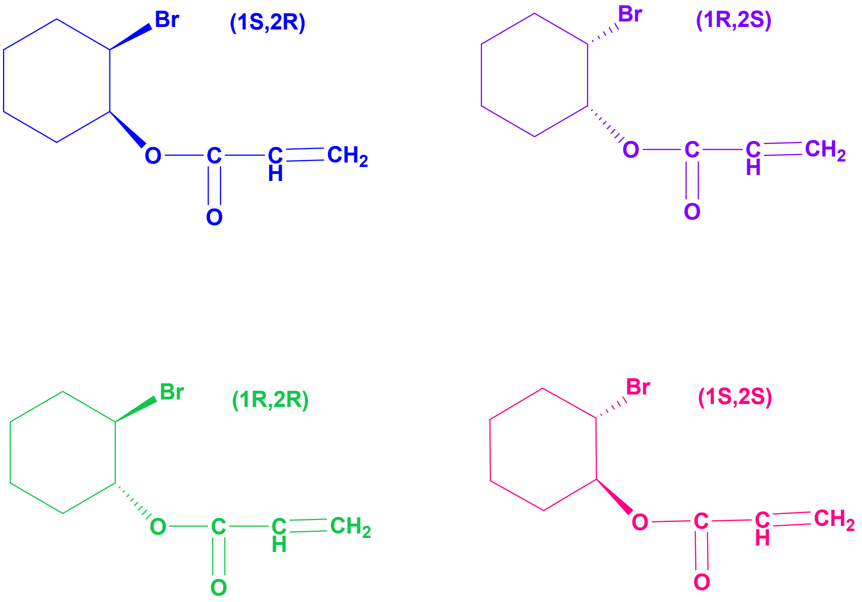

Figure S1. Stereoisomers of monomer M1.

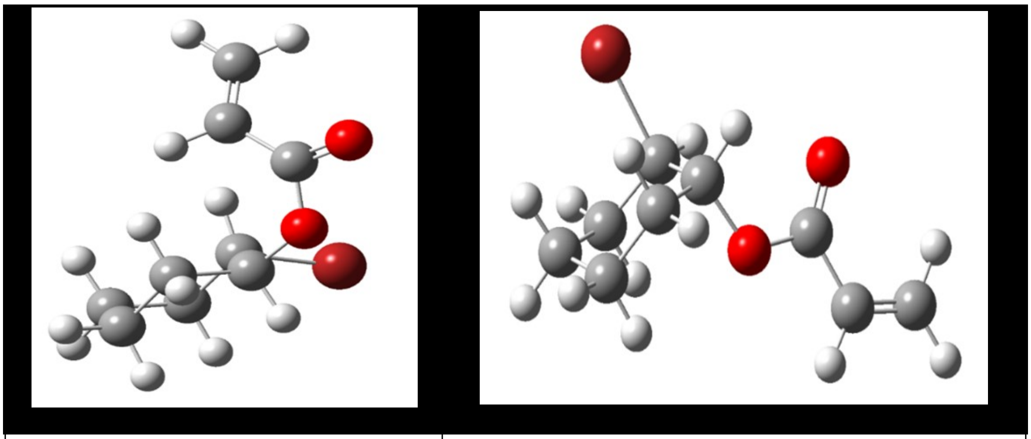

**Cis bromoacrylate E = -3072,652 hartree**	**Trans bromoacrylate E = -3072,676 hartree**

Figure S2. The most stable conformer of the bromoacrylate molecule.

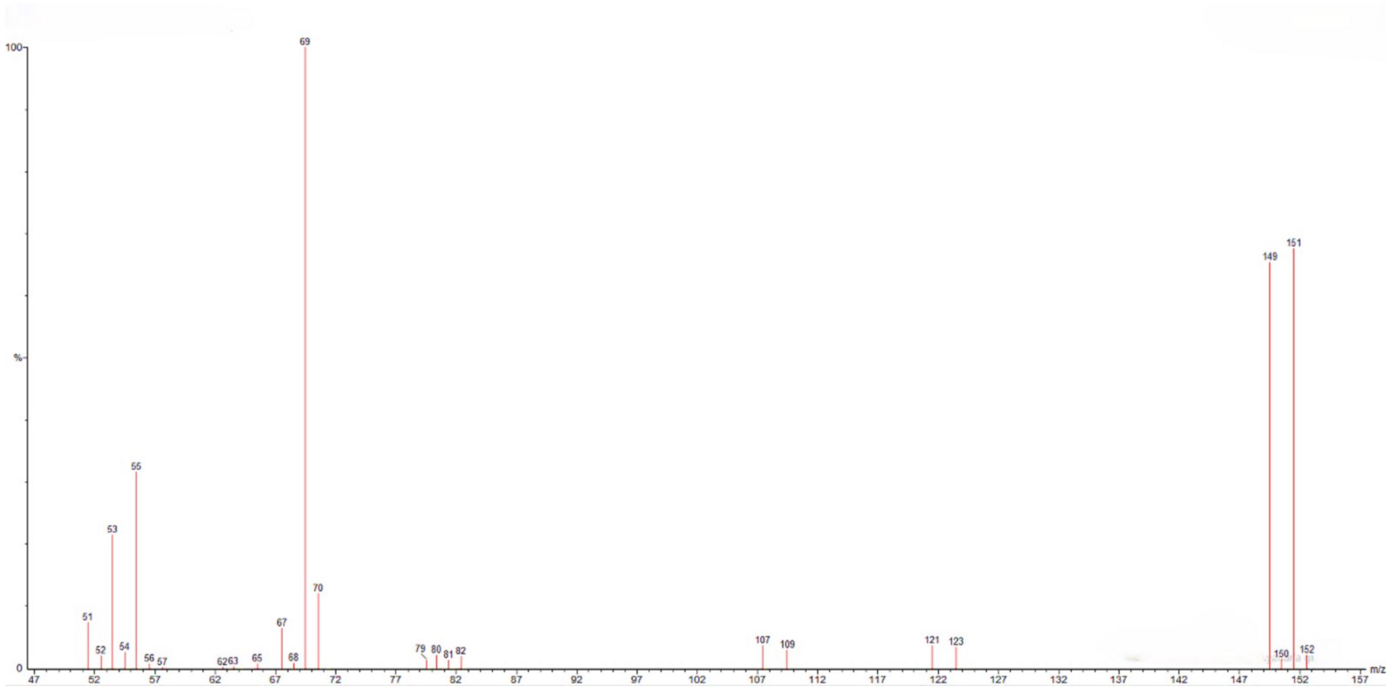

Figure S3. GC-MS spectrum of monomer.

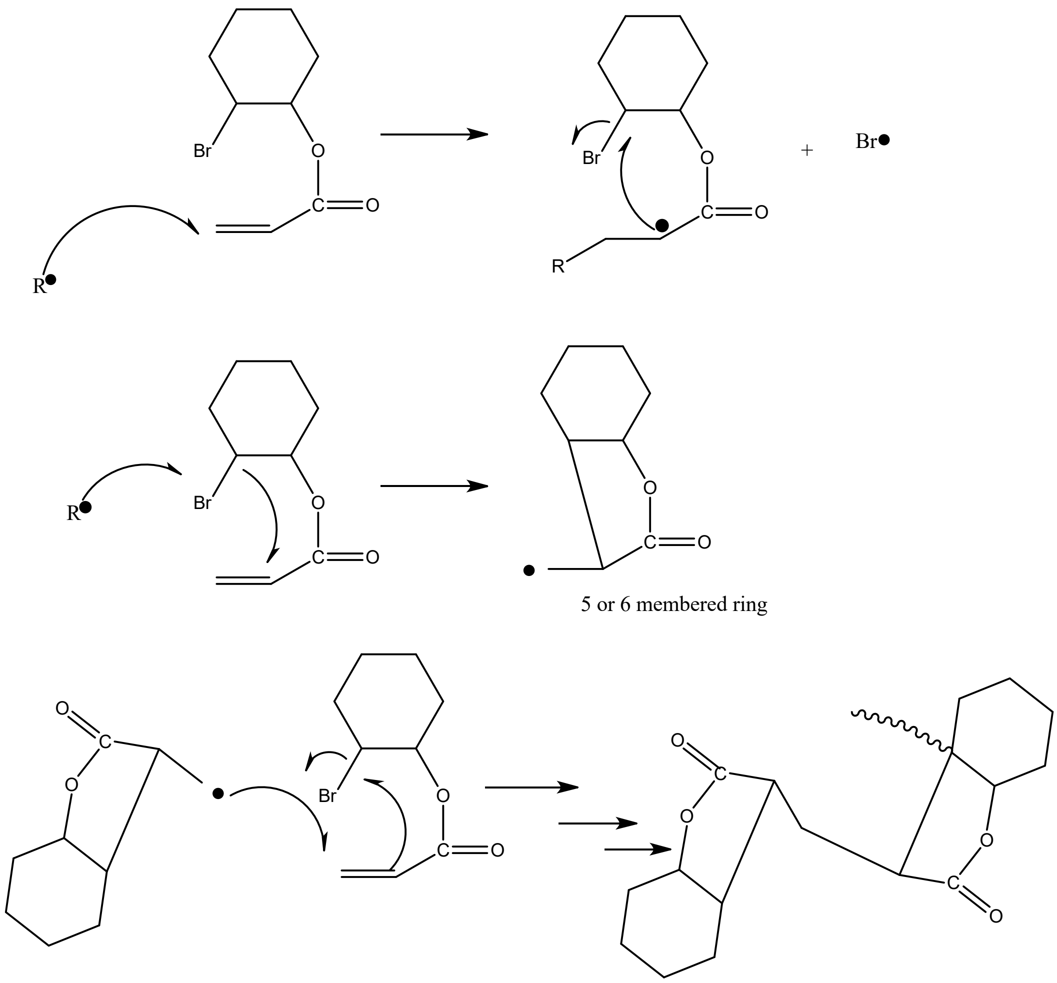

Scheme S1. Proposed intramolecular cyclopolymerization route

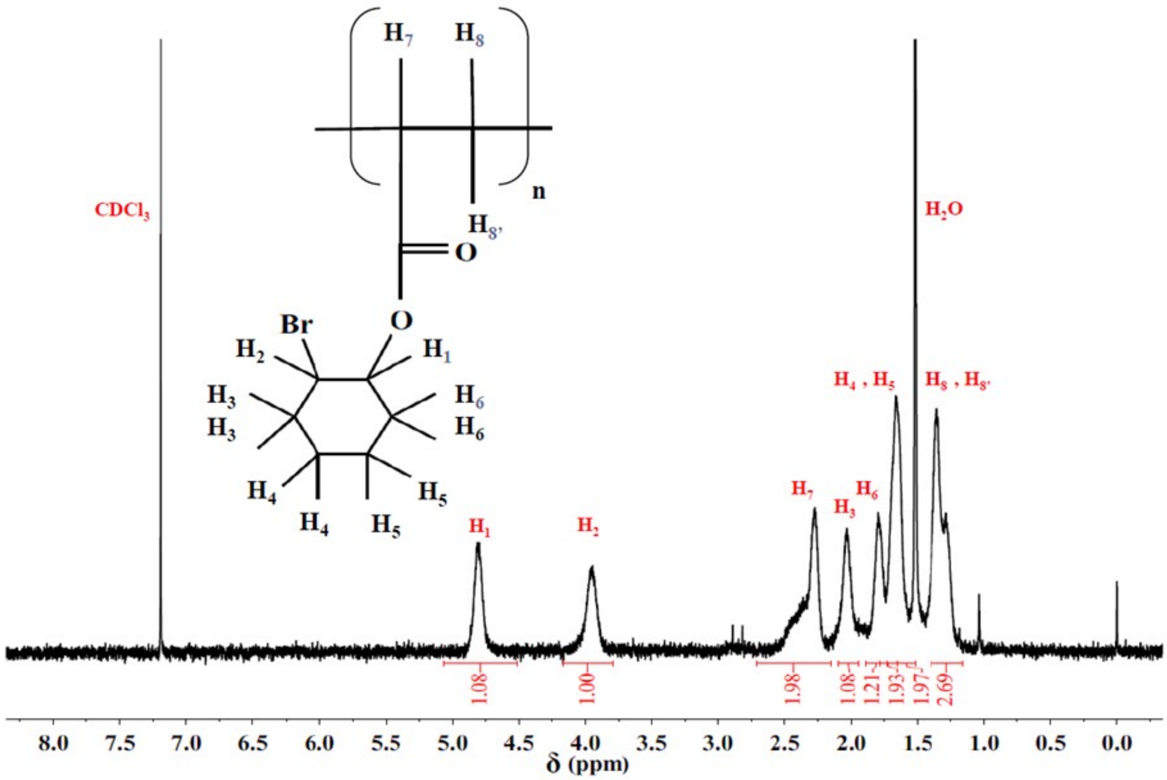

Figure S4. ^{1}H NMR spectrum of P2.

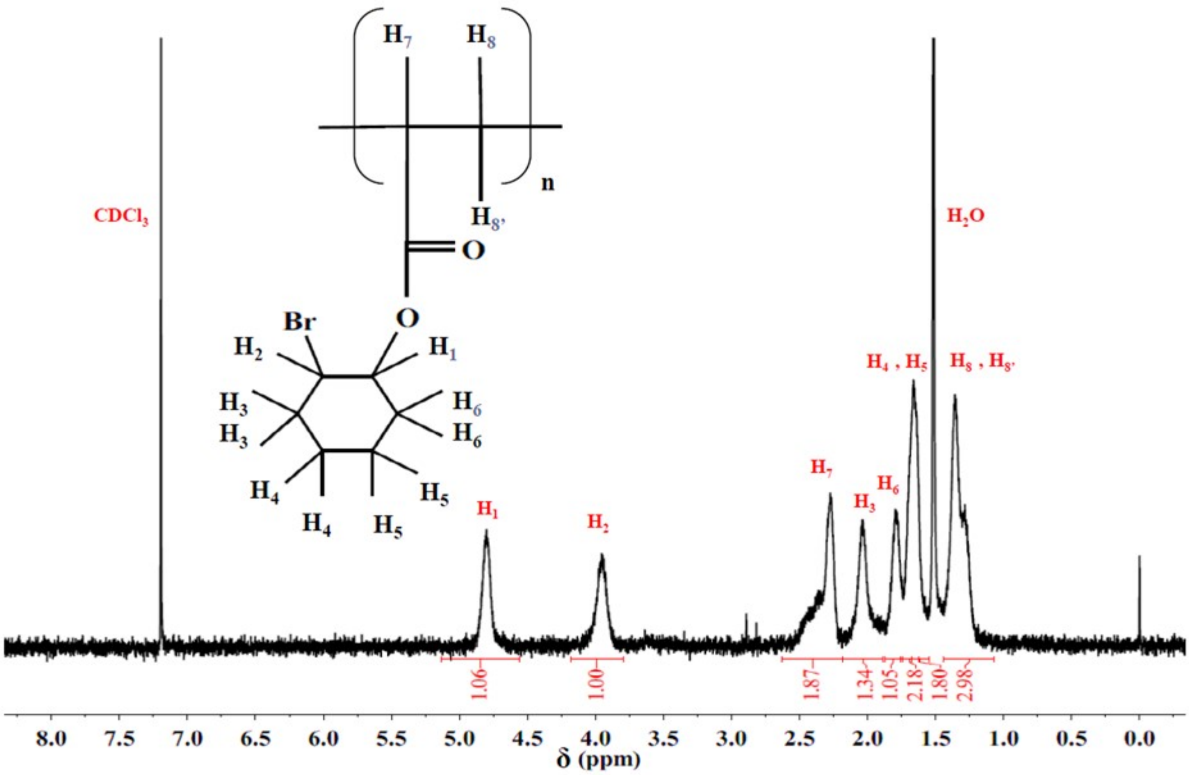

Figure S5. ^{1}H NMR spectrum of the P3.

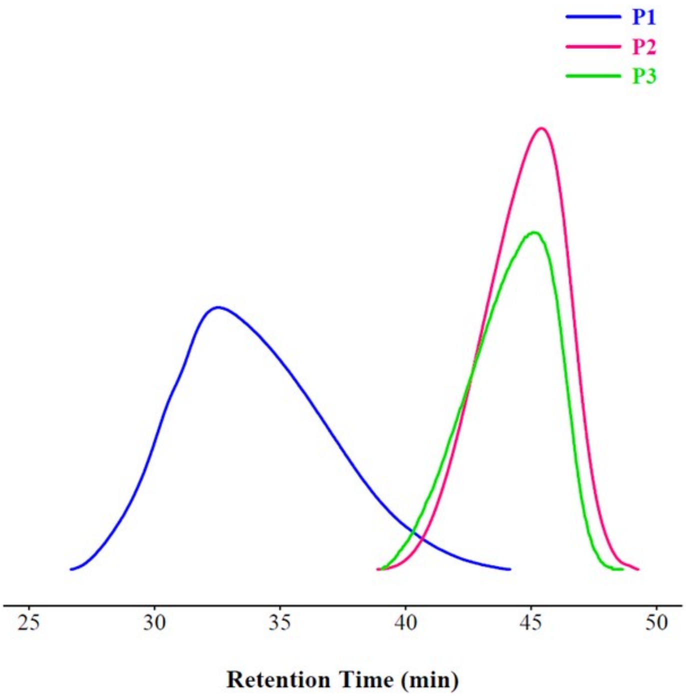

Figure S6. GPC graph of polymers.

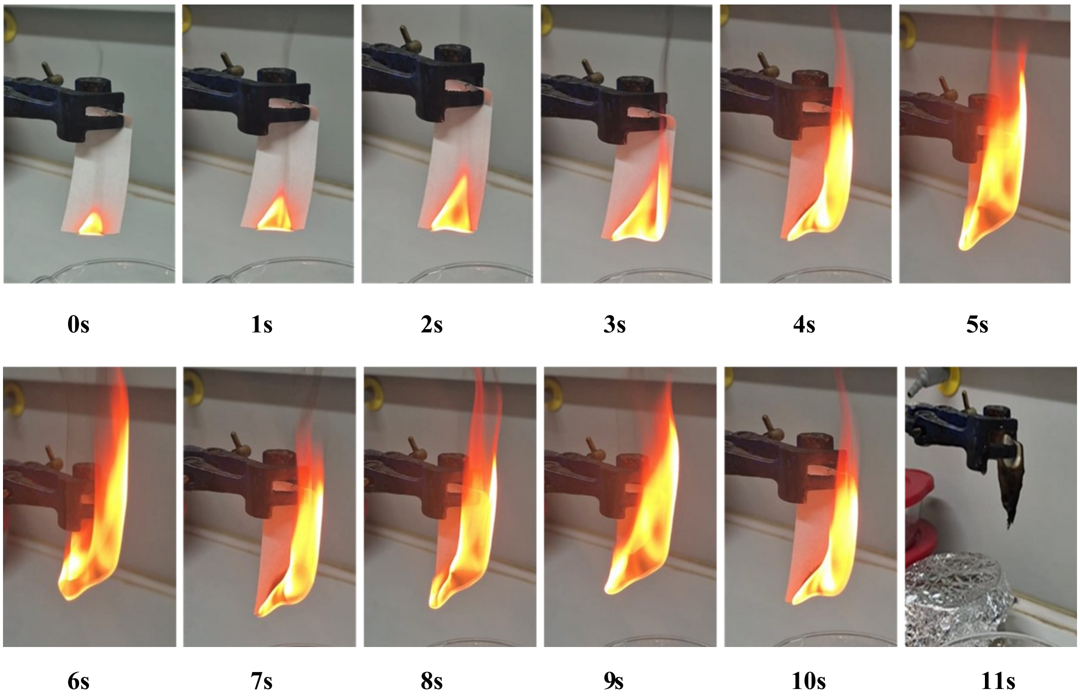

Figure S7. Photographs of the burn test conducted on uncoated filter paper (a), filter paper coated with polymethylacrylate (b) and P1 (c).

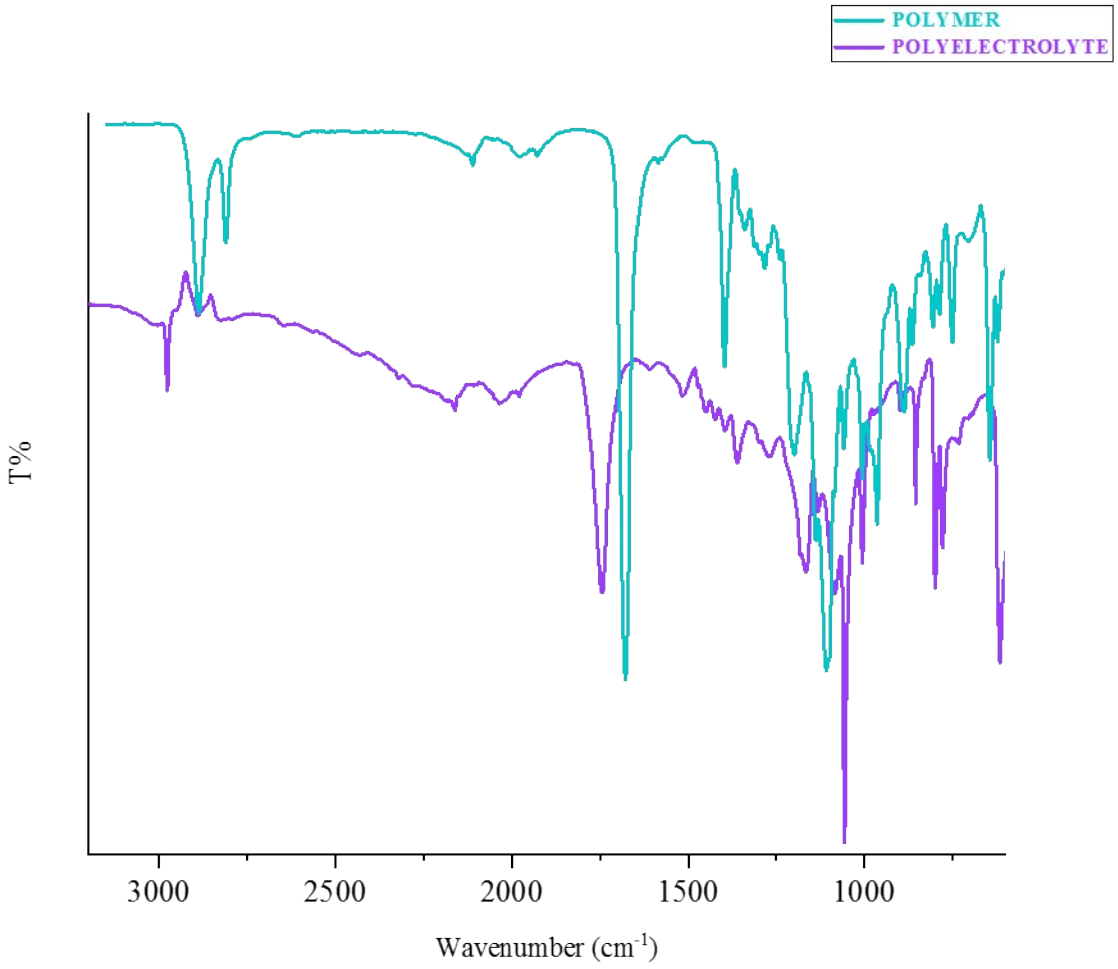

Figure S8. FTIR spectra of polymer (upper) and quaternized (DABCO derivative)

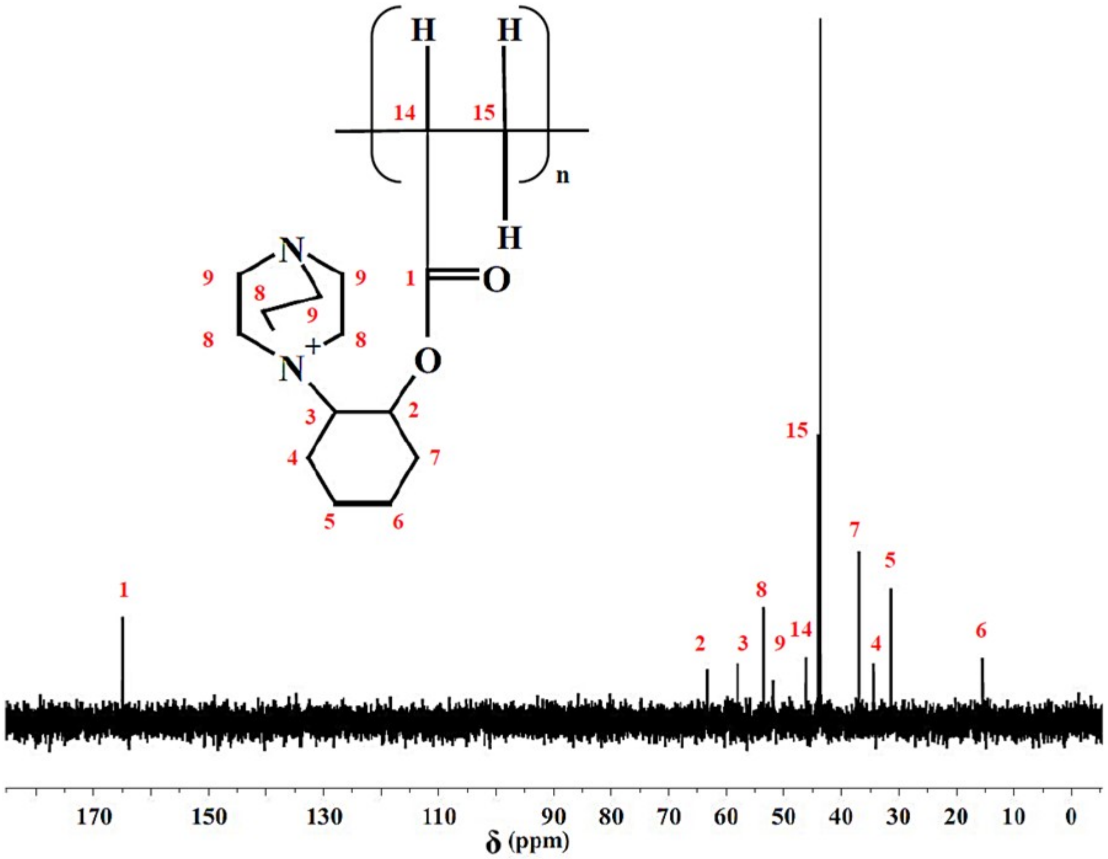

Figure S9. ^{13}C NMR spectrum of the DABCO-functionalized polymer

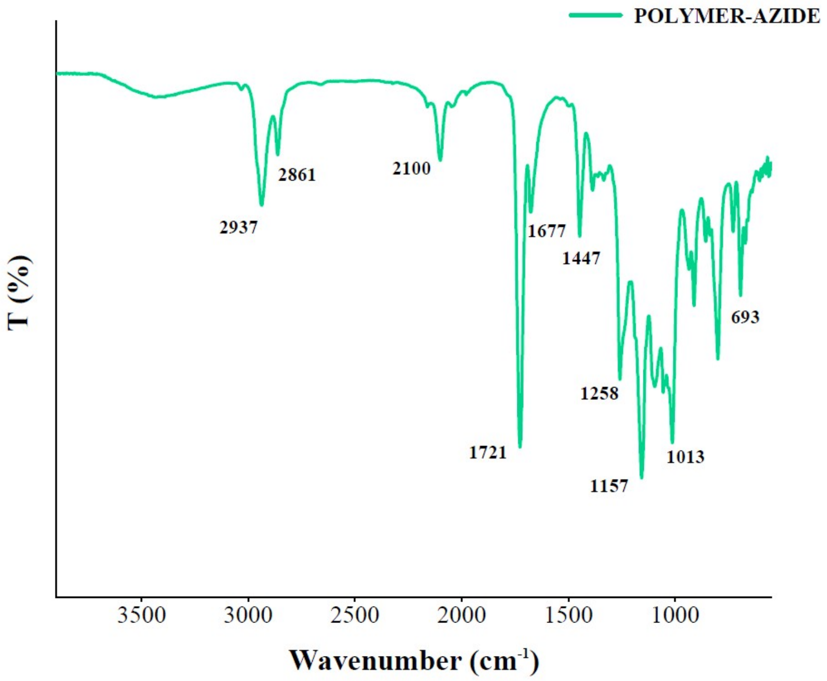

Figure S10. FTIR spectrum of polymer-azide adduct.

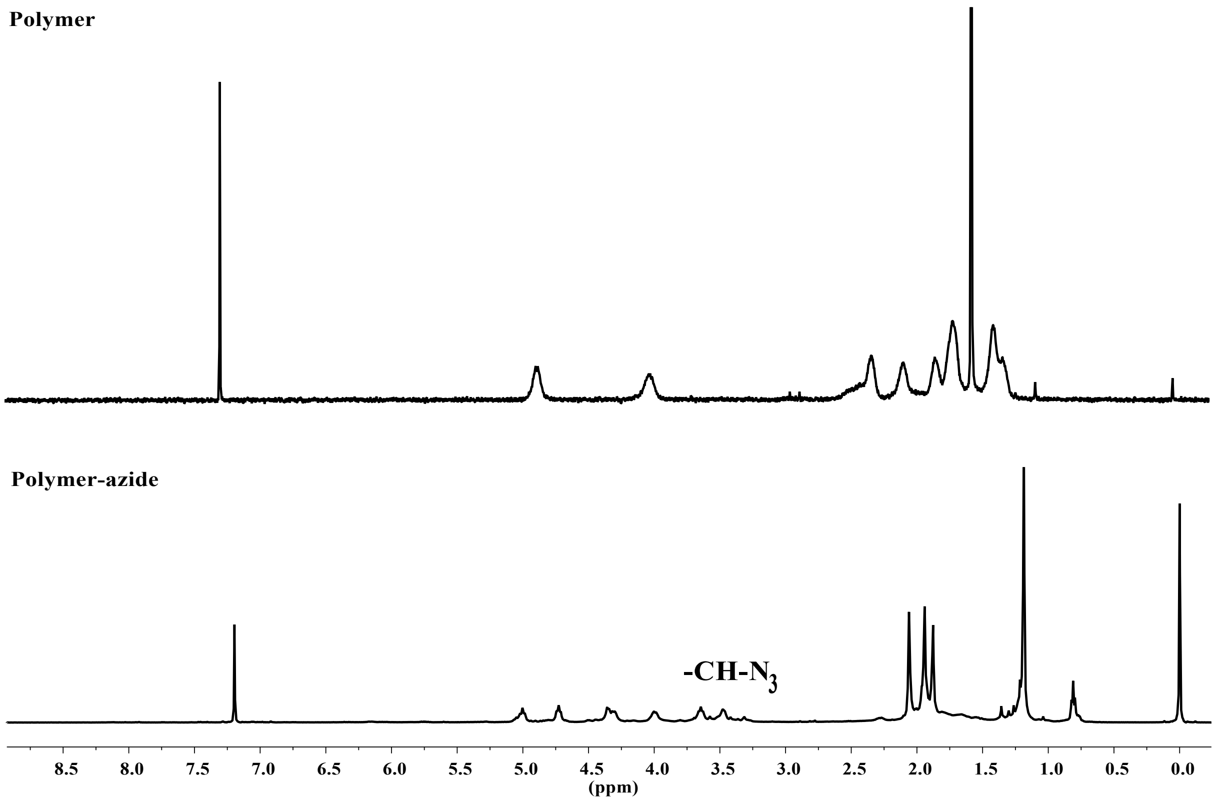

Figure S11. ^{1}H NMR spectra of the azide-functionalized polymer and polymer.
